# Rush Medical College

**Published:** 1859-03

**Authors:** 


### RUSH MEDICAL COLLEGE.

It will be observed by the notice in this number of the *Journal*, that Professors Johnson, Davis, and Byford have withdrawn from the faculty of Rush Medical College. Of the reasons which have induced them to take this step we do not propose to speak at present.

In behalf of the remaining faculty, we deem it proper to say, that the organization and efficiency of the college will not suffer any diminution, and that the means of teaching, for the future, will be rather increased than lessened, by the effect of this withdrawal.

The selections for filling the vacant chairs have not yet been made, but, from the eminent and capable gentlemen who have already intimated their willingness to accept, the Board of Trustees will only have the embarrassment of a choice. For clinical teaching, the college dispensary will be continued during the entire year, students will have access to the Marine Hospital during the winter session, and whatever other facilities for hospital instruction the city may afford, will be open to them.

The present plan of conducting the course of winter instruction will not be essentially changed, unless by concert with other medical schools. A preparatory department will be organized for the summer term, in which the active and younger members of the profession of the city will be enlisted. Good instruction throughout the year will thus be secured.

Pointing with satisfaction to her history and her eminence, Rush Medical College will not falter nor swerve from that course of usefulness which she has hitherto preserved.

☞ The present proprietor of the *Chicago Medical Journal* is desirous of securing the services of an assistant editor. To one competent and with some experience, the situation would be advantageous.

